# Violences, suicidal behaviour, and non-suicidal self-injury in child development: findings from a Brazilian cohort

**DOI:** 10.1186/s13034-025-00961-x

**Published:** 2025-10-31

**Authors:** Orli Carvalho da Silva Filho, Joviana Quintes Avanci, Thiago de Oliveira Pires, Raquel de Vasconcellos Carvalhaes Oliveira, Simone Gonçalves de Assis

**Affiliations:** 1 National Institute of Women, Children and Adolescents Health Fernandes Figueira/Oswaldo Cruz Foundation (IFF/Fiocruz), Av Rui Barbosa, 716. Flamengo, Rio de Janeiro, RJ 22250-020 Brazil; 2 National School of Public Health/Oswaldo Cruz Foundation (Ensp/Fiocruz), Rio de Janeiro, RJ Brazil; 3 Adventist University Center of São Paulo, São Paulo, Brazil; 4 National Institute of Infectious Diseases/Oswaldo Cruz Foundation (INI/Fiocruz), Rio de Janeiro, RJ Brazil

**Keywords:** Suicide, Self-injurious behavior, Violence, Child development, Adolescent, Youth

## Abstract

**Background:**

Violence and mental health have a large proportion within the global burden of disease for children and youth, especially with the growing magnitude of suicidal behaviour and non-suicidal self-injury. This longitudinal study examined for the effects of physical and psychological family and community violence during childhood, adolescence and youth and the emergence of suicidal behaviour and non-suicidal self-injury among young people.

**Methods:**

129 participants, from a cohort of 500 students (mean age 8 years, SD 1.2) sampled at schools in a Brazilian metropolis (2005), were followed up for 16 years (2006, 2008, 2012, 2021). Suicidal behaviour/non-suicidal self-injury by the youth was the dependent variable assessed at the fifth wave of the study by Adult Self Report/Achenbach System of Empirically Based Assessment (ASR/ASEBA) and Youth Risk Behavior Survey Scale (YRBSS). The independent variables were 193 questions on violence (Conflict Tactics Scale - CTS - and “Things I have seen and heard”), which were assessed longitudinally. Three groups of violence (physical family violence, psychological family violence and community violence), plus the three stages of development studied (childhood, adolescence and youth), resulted in nine violence events, which were examined descriptively and input to structural equation modelling.

**Results:**

The outcome was found in 37% of the participants, combining all time points. The forms of violence that occurred in childhood, adolescence and youth were, respectively: physical family violence: 84.5%, 14% and 15.5%; psychological family violence: 76.7%, 3.9% and 76.7%; and community violence: 18%, 12.4% and 40.3%. The modelling revealed a network between types of violence: community and psychological (0.348; *p* < 0.001) in childhood; community and psychological (0.302; *p* < 0.001) and physical and psychological (0.374; *p* = 0.001) in youth; and among all types of violence in adolescence. Regarding the outcome, the following factors were prominent: psychological family violence in childhood (0.656; *p* = 0.006), community violence in adolescence (0.517; *p* < 0.001) and psychological family violence in youth (0.398; *p* < 0.001).

**Conclusions:**

The study highlighted the effect of different forms of violence longitudinally with suicidal behaviour and non-suicidal self-injury in youth, underlining the value of preventing violence as an important vector in related intervention.

## Background

The study of different forms of violence has gained prominence in relation to childhood, adolescent and youth morbidity and mortality [1]. That prominence goes beyond the direct impact of deaths from external causes, and to include the influence of violence on paediatric mental health [2–4]. This influence, linked to severe psychological distress and limited access to care, is associated with adverse outcomes such as suicidal behavior and non-suicidal self-injury [5–8].

Suicidal behavior (SB) is a *continuum* involving ideation, planning, suicide attempts and suicide [9, 10]. Self-injury is a polymorphic phenomenon in which self-inflicted violence may occur with or without suicidal intent. It is defined as non-suicidal self-injury (NSSI) when the self-harming behavior is not accompanied by a desire to die [9, 10]. These phenomena are occurring at an order of magnitude that signals suicide to be a public health concern for global youth [4, 6]. In several countries, suicide is a leading cause of death in adolescents and young people and can be considered a public health crisis calling for efforts to increase understanding and prevention [2, 11, 12].

For about five decades, the Health Sector has argued that violence should not be confined to the legal domain or law enforcement alone [13, 14]. The WHO´s *World Report on Violence and Health* [14] adopts the bio-ecological model of development, which views human development as shaped by complex interactional systems influencing care and protection against violence throughout life, particularly in vulnerable contexts [15, 16].

Interest in the repercussions of violence and the importance of promoting healthy human development [17, 18] has encouraged learning more about adverse childhood experiences (ACEs). ACEs refer to experiences of violence or adversity occurring during childhood and adolescence, which have the potential to be traumatic and detrimental to psychological development and mental health [17, 19]. The ACE construct encompasses a range of harmful experiences, including abuse, neglect, and/or household dysfunction [20]. ACEs have thus come to constitute the possibility of providing instruments for studying trauma and violence in childhood and adolescence [20, 21] enabling a methodological pathway to understand how different forms of violence act in early life and their impact in the present and future [20].

When examined over a life course, evidence links emotional suffering to violence and ACEs [7]. Kessler et al. [22] estimated a 29.8% population attributable risk (PAR) of such violence for nonspecific mental disorders. Poor family functioning predicts greater impact from ACEs [22, 24], and Teicher and Samson [23] found a PAR of 45% when psychopathology occurs in childhood. Jewett et al. [24] and Thompson et al. [25] reported associations between ACEs and suicidal behaviour across the life course, without assessing mental disorders.

Although biomedical literature indicates a robust association between mental disorders and suicide [26], no precise understanding yet exists of the developmental trajectories linking childhood adversities to various health outcomes [6, 8, 11]. Teicher et al. [23, 27] argued that the main neuro-biological alterations found in psychological disorders are adaptations resulting from ACEs and violence. This argument hypothesizes that violence may impact developmental trajectories more significantly than a psychiatric diagnosis. On that basis, psychological suffering categorised as disorder is regarded as an eco-phenotype resulting from a violent environment [27].

Violent environments affect various health domains. ACEs have cumulative, long-term impacts with a tendency to repeat [21] and show a bidirectional relationship with family functioning, where dysfunction worsens outcomes [23, 24]. Violence against children and adolescents often occurs with other vulnerabilities, amplifying effects through capillarity and crystallisation [21, 24, 28, 29]. Regarding developmental pathways and sensitivities, the timing, type, and severity of violent exposure are key in the onset of adverse mental health outcomes, modulating individual susceptibility, resilience, and protective factors [20, 30, 31]. As Ports et al. [28], preventing suicidality is urgent to preventing violence.

Longitudinal studies of mental health are scarcer than cross-sectional studies, and even more incipient are those addressing developmental trajectories [25, 30]. Accordingly, taking a cohort (2005–2021) that followed schoolchildren in a Brazilian metropolis through to late adolescence, it was decided to study adverse mental health outcomes and the adversities experienced, by identifying specific categories of violence prevalent in that community [32]. This longitudinal study examined for the effects of family (physical and psychological) and community violence occurring during childhood, adolescence and youth (2005–2021) and the emergence of suicidal behaviour and non-suicidal self-injury in youth (2021).

## Methods

This article draws on a prospective study of a cohort of children sampled in 2005 at public schools in a municipality in southeast Brazil with a population in 2022 of 896,744 (22,6% 0–19 years old) and notable for strong social inequality, precarious infrastructure and high levels of poverty and violence. Worsening these conditions, the population’s low level of education offers few opportunities for skilled employment, encouraging commuting migration. It used data collected at the baseline and first data collection wave (2005) and at another four follow-up waves (2006, 2008, 2012 and 2021). The study data thus cover the stages of the participants’ childhood, adolescence and part of their youth.

All stages of this study were approved by the research ethics committee of the Escola Nacional de Saúde Pública/Fundação Oswaldo Cruz (CAAE 18723119.0.0000.5240). All participants signed declarations of free and informed consent.

### Study population

The longitudinal sampling plan was based on a population of 6,589 pupils in the second year of lower secondary school in municipal public schools in 2005. Sampling selection occurred in three stages: 25 schools (in proportion to size), classes (simple random) and 10 pupils per class (simple random), for a total sample of 500 pupils (50% proportion, 98.02% level of confidence and 5% relative error). In 2005, 231 children were replaced from the original list, primarily due to errors in the official class rosters, as well as absences caused by illness or the lack of a guardian on scheduled days, despite three attempts. Sampling losses in 2006, 2008, 2012 and 2021 were, respectively, 5.6%, 10.6%, 24.2% and 74.2% of participants in relation to the initial sample. Sample at the end of the study resulted in a higher proportion of women and a lower proportion of white participants compared to the baseline sample (Table 1).

**Table 1:** Sociodemographic profile of cohort participants (Waves 1 and 5)

	**2005 (** ***N*** ** = 500)**	**2021 (** ***N*** ** = 129)**
**Mean age (years/SD)**	8.0 (SD 1.2)	23.8 (SD 1.1)
Sex (%) *Male* *Female*	51.6% (48.5% - 54.7%) 48.4% (45.3% − 51,5%)	41.9% (34.3% − 49.7%) 58.1% (50.3% − 65.7%)
**Colour (% as self-declared by the family/2005 and young person/2021)** *White* *Black + Mixed* *Yellow / Indigenous*	33.5% (30.0% − 37.7%) 65.9% (62.3–70,0%) 0.6%	22.8% (17.2% − 29.6%) 75.6% (68.0% − 81.9%) 1.6% (0.4% − 6.6%)
**Family (%)** *Lives with*: *Parents or stepparents* *Grandparents*,* uncles*,* aunts or other relatives* *Partner* *Alone* *Has children*	95.8% (93.2% − 97.4%) 4.2% (2.6% − 6.8%) ---	53.8% (40.3% − 66.8%) 36.0% (25.7% − 47.8%) 57.2% (47.7% − 66.2%) 5.5% (2.6% − 11.1%) 36.2% (26.5% − 47.3%)
**Schooling (%)** *Age-appropriate – % two or more years behind in 2005 (pupils from age 9 years on)* *In school to what age?*	25.4% (21.1% − 30.2%) ----	16.3% (11.0% − 23.5%) *- Lower secondary (complete and incomplete*) 22.6% (16.5% − 30.1%) *- Upper secondary (complete and incomplete*) 66.5% (56.4% − 75.2%) *- Higher (complete and incomplete)* 10.9% (6.0% − 19.2%)
**Work** *Paid work (%)* *Unpaid work* Does not work	----	58.3% (51.6% − 64.6%) 00% (10.1% − 21,7%) 26.8% (20.8% − 33,7%)

Source: the authors (2024)

SD – standard deviation

CI – Confidence Interval 95%

### Measures

The dependent variable of interest was suicidal behaviour/non-suicidal self-injury (SB/NSSI); it is a dichotomous variable and was identified using a questionnaire self-applied in 2021, by at least one positive response to any of the following questions: “Have you talked or thought about killing yourself in the past six months?”; “Have you deliberately harmed yourself or tried to kill yourself in the past six months?”; “Have you thought seriously of killing yourself in the past year?”; “Have you planned to kill yourself in the past year?”; “Have you attempted suicide in the past year?”; and “Have you attempted suicide before the past year?”. The first two questions form part of the Adult Self Report (ASR), an instrument of the Achenbach System of Empirically Based Assessment scale (ASEBA) [33], continuing with the scale already used over the course of the cohort. The other questions are from the Youth Risk Behavior Survey Scale (YRBSS), which was used only in 2021 [34].

Despite the divergence in the literature as to whether the suicide spectrum comprises all forms of self-harm [9, 35], the outcome of interest to the study included: suicidal ideation, attempted suicide and non-suicidal self-injury. In that way, suicidality was conceived overall as proposed by the Integrated Motivational-Volitional (IMV) Model of Suicidal Behaviour [36, 37] and as in previous studies of this same cohort [10]. The aim was to explore potential similarities among various expressions of self-inflicted violence, with an emphasis on its different effects.

The violence variables were formulated from items taken from two scales applied over the years: the Conflict Tactics Scale (CTS) [38], which measures physical and psychological violence in the family and “Things I Have Seen and Heard” [39], which measures family and community violence. Both are fully standardized instruments, adapted/validated for Portuguese [40, 41], and were systematically administered to the same individuals across the waves - answered by the caregiver during childhood and by the young person from adolescence onward. Few other questions present over the years were incorporated. Considering that the children were of school age at the beginning of the cohort, some variables related to early childhood were retrospectively reported by caregivers during the first wave.

In the five waves, 193 variables that measure different forms of violence (80 in the family psychology context, 70 in the physical family context and 43 in the community context) were tabulated against the dependent variable SB/NSSI. Only the 32 with statistical significance (*p* < 0.10) were retained in subsequent analyses and are described below, forming three violence groupings.

Physical family violence involved father and mother (or guardians) acting separately, at moments of conflict over the course of development, in the following ways: “pushed, grabbed or shoved him/her”, “slapped or spanked him/her”, “hit or tried to hit him/her with something”, “threw something at him/her” or “kicked, bit, or hit him/her with a fist”. Youth were asked whether the partner “threw something at you”, “pushed, grabbed or shoved you” or “slapped or spanked you”. Of the 11 selected variables, eight referred to violence caused by the mother and three, by the partner.

Psychological family violence - over the course of development, at moments of conflict, father and mother (or guardians), acted separately in the following ways: “sulked or refused to talk about an issue”, “did or said something to spite him/her”, “stomped out of the room, house or yard”, “threw, smashed, hit or kicked something”, insulted or swore at him/her”, “cried” or “threw, smashed, hit or kicked something”. The young people retained in the study were asked: “whether they saw adults in the house shout at each other” or whether “the partner insulted or swore at you”. Of the selected 16 variables, nine reflected violence by the mother, four by the father, one by both parents in adult life, one by partners and one from witnessing violence between parents.

Community violence (5 variables) – whether, in the community environment, the participant had: suffered humiliation in the school or community, “seen somebody beat up”, “seen somebody get shot”, “their house broken into” or suffered sexual harassment/abuse at school, in the community or by strangers.

Each of the items that make up these three groups of variables was transformed into “present or absent”, thus behaving as a “violent event”. Subsequently, these events were added up, by the three categories of violence and by the three stages of development at which they occurred. The stages were considered to be: childhood, from 6 to 9 years of age, adolescents, from 10 to 19 years of age, and youth, from 20 years onwards. Note that five variables retained at this stage of the study, relating to physical and psychological family violence perpetrated by the mother, referred not only to the year prior to the study (as is usual with the CTS scale), but to the child’s lifetime, from birth up to the first data collection wave in 2005, with a view to including information about violence in early childhood.

In this way, at a single wave, records of the events of violence for the total sample could be distributed by different stages, depending on the participant’s age at the start of the study; that is, by reference not to the data collection year, but to the stage of development. This enabled participants to be grouped by their developmental stage and not simply by the time at which they responded to the questionnaire.

### Analysis: descriptive stage and structural equation modelling

The three groups of violence (physical family violence, psychological family violence and community violence) and three stages of life (childhood, adolescence and youth) gave the nine categories of violence retained in the analysis as instruments for the study of violence in the lives of the 129 young people interviewed in 2021: psychological family violence in childhood (PsyFV_C), psychological family violence in adolescence (PsyFV_A), psychological family violence in youth (PsyFV_Y), physical family violence in childhood (PhyFV_C), physical family violence in adolescence (PhyFV_A), physical family violence in youth (PhyFV_Y), community violence in childhood (ComV_C), community violence in adolescence (ComV_A) and community violence in youth (ComV_Y).

First, the nine categories of violence were analysed descriptively. At each stage of life, each category was considered positive for violence if at least one violent event was present, allowing the distribution of violent events within the cohort to be assessed. The frequencies in each category allowed a sum of violent items to be transposed to structural equation modelling, where relations among these categories, as well as with the SB/NSSI outcome, were evaluated by way of path coefficients [39, 40].

Structural equation modelling, a family of multivariate statistical modelling techniques and causal inference methods, returned the strength of relations among the theoretical constructs used here [42, 43]: between each category of violence and the outcome of interest, as well as among the categories of violence themselves. Sample weighting was included in that analysis to correct the specific estimates. At first, an attempt was made to include an interval estimate correction plan, but the model displayed a problem of convergence due to the small number of individuals per school. The model outcome was binary, employing the probit link function. The R statistical package, version 4.3.0 [44], was utilized. The lavaan.survey package was applied for the analysis of the structural model with complex sampling. For the remaining procedures, the survey package was adopted [42]. The model’s goodness-of-fit was monitored using the global Root Mean Square Error of Approximation (RMSEA), Standardized Root Mean Square Residual (SRMR), and Comparative Fit Index (CFI) indices. As a criterion for model acceptability, the following thresholds were adopted: RMSEA ≤ 0.06, SRMR ≤ 0.08, and CFI ≥ 0.95 [45].

**Fig. 1:**
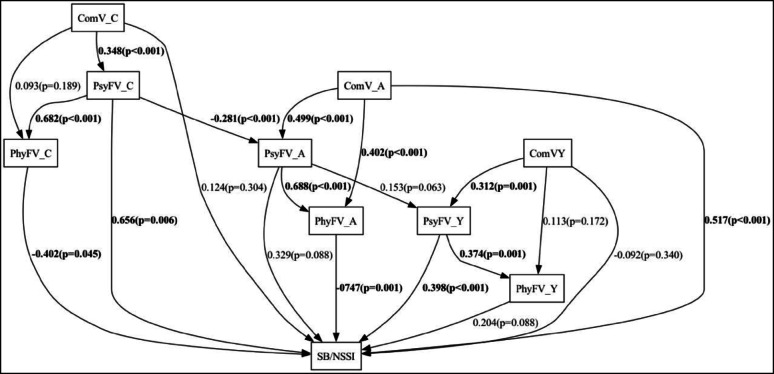
Structural equations model among the nine categories of violence and suicidal behaviour/non-suicidal self-injury. Source: the authors (2024). Legend: SB/NSSI: suicidal behaviour/non-suicidal self-injury; PsyFV_C: psychological family violence in childhood; PsyFV_A: psychological family violence in adolescence; PsyFV_Y: psychological family violence in youth; PhyFV_C: physical family violence in childhood; PhyFV_A: physical family violence in adolescence; PhyFV_Y: physical family violence in youth; ComV_C: community violence in childhood; ComV_A: community violence in adolescence; ComV_Y: community violence in youth. RMSEA: 0,114 (0,079 – 0,150); SRMR: 0,076; CFI: 0,840. Significant associations are highlighted in bold

The theoretical model for the occurrence of suicidal behavior in youth assumes that: family and community violence are frequent and accompany the developmental trajectory [17]; both types of violence feed of each other; that psychological and physical violence commonly occur together and also intensify each other [22]; that family violence in childhood tends to be higher than in adolescence, and the opposite is true for community violence [14]. The coefficients obtained in the structural equation model, and the Goodness of Fit indices were added in Fig. 1, which represents the theoretical model [44]. Figure 1 presents the paths between the nine categories of violence studied (the independent variables) and suicidal behaviour/non-suicidal self-injury (the dependent variable) and the connections among these nine categories themselves. Expressed as a graph in this way, the coefficients can represent relations among the theoretical constructs underpinning this study.

**Fig. 2:**
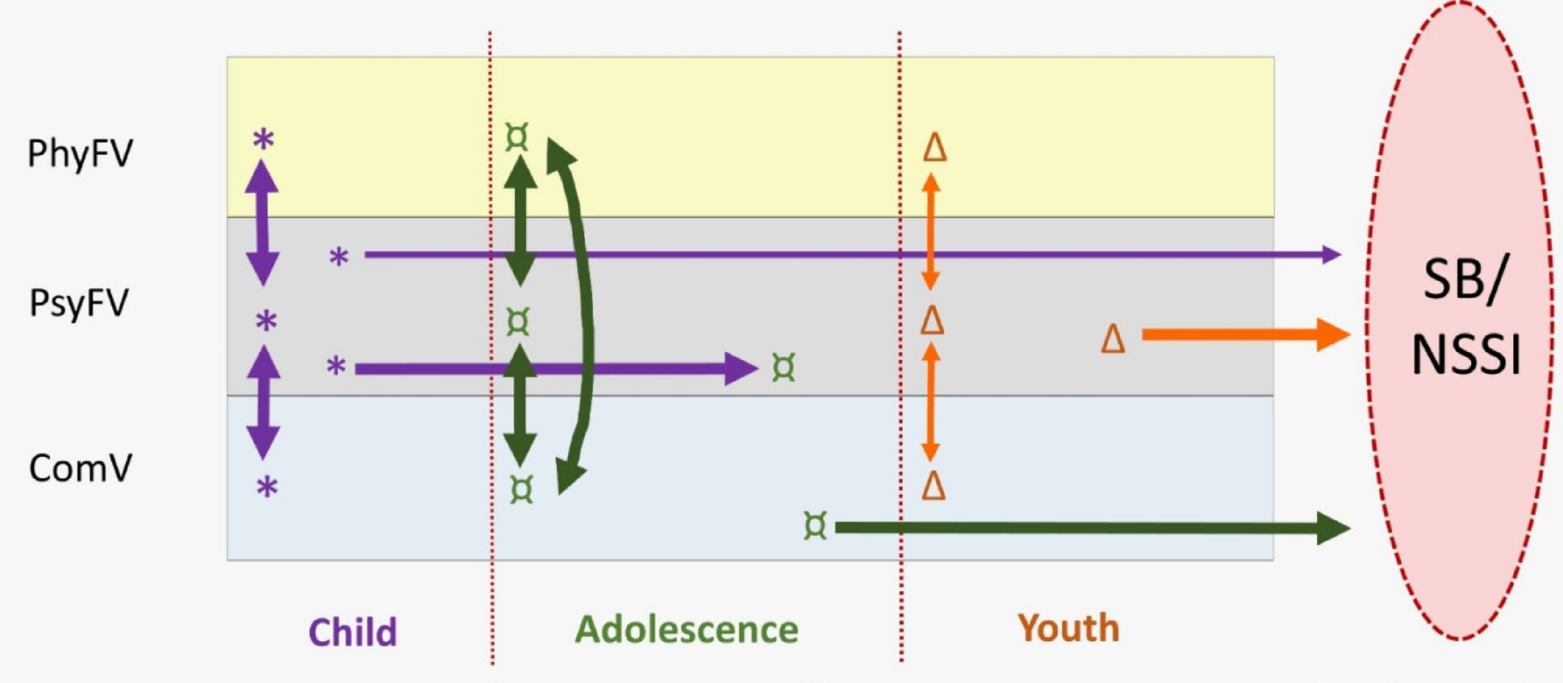
Diagram representing structural equations model relating the nine categories of violence and suicidal behaviour/non-suicidal self-injury. Source: The authors (2024). Legend: SB/NSSI: suicidal behaviour/non-suicidal self-injury; PhyFV: physical family violence; PsyFV: psychological family violence; ComV: community violence; * = category of violence present in the childhood developmental stage; ¤ = category of violence present in the adolescence developmental stage; Δ = category of violence present in the youth developmental stage. Arrow thickness is proportional to the significance of the trajectories and connections between variables

As this was a complex model, a simplified diagrammatic representation was proposed (Fig. 2). This involved selecting the categories whose paths were associated directly and most significantly with the outcome, as indicated by p-value (*p* < 0.05), even though, in the structural equation modelling, that measure also had to be interpreted in the light of the path coefficients. The priority was to organise in such a way that it was easier to visualise through the three stages of life, without however disregarding the complex web of effects among the forms of violence. The thickness of the arrows is proportional to the significance of the paths they represent, thus portraying the strength of the effects graphically. The arrows in the figure are of three thicknesses: the thickest indicate the strongest significance (*p* < 0.001); medium thickness indicates a significance of between p-values 0.001 and 0.006; and the thinnest indicates the least significant among those selected (*p* = 0.045).

## Results

The sociodemographic profile of the participants in 2005 and in 2021 is shown in Table 1. The 25.8% of the participants who continued in the study in 2021 are representative of the baseline sample (2005), which enables comparisons to be made along the course of the cohort. The population was predominantly black and mixed, and academically backward. Longitudinally, there was a slight predominance of female participants and a reduction in the frequency of participants living with parents or stepparents.

In 2021, of the 129 young people who continued in the cohort, 11.7% reported suicidal ideation in the past year and 7%, prior to the past year; and 4% reported attempting suicide once in the past year; 1.6%, twice or three times in the past year; and 4.9%, prior to the past year. The frequency of positive responses to the item “Have you deliberately injured yourself or attempted suicide in the past six months?” was 13.2%.

Occurrences of SB/NSSI tended to increase over the course of the study, particularly between the fourth and fifth waves. Table 2 shows the frequencies of the outcome (SB/SNNI) of young people interviewed at the last wave of the cohort.

**Table 2:** Frequency (CI) of SB/NSSI in lives of young people interviewed at the last wave of the cohort (*N* = 129)

	How many times?	%	Confidence Interval 5%
SB/NSSI	At least once at some point of the study	37.0	29.2–46.1
	Once	16.3	11.8–22.1
	Twice	11.6	6.2–20.8
	Three times	8.6	3.7–18.4
	Four times	0.8	0.1–5.6

Source: the authors (2024)

CI – Confidence Interval 95%

Table 3 shows the frequencies of the three types of violent events over the course of the three developmental stages. Physical family violence (84.5%) and psychological family violence (76.7%) stood out in childhood, while youth experienced mostly psychological family violence (76.7%) and community violence (40.3%).

**Table 3:** Frequency (CI) of violent events in lives of young people interviewed at the last wave of the cohort, by stages of development (*N* = 129)

	Childhood	Adolescence	Youth
Family and community violence, scores > 0			
Physical family violence	84.5% (76.9%- 89.9%)	13.9% (9.8% − 19.4%)	15.5% (11.3% − 20.9%)
Psychological family violence	76.8% (69.9% − 82.5%)	3.9% (1.8% - 8.3%)	76.8% (69.7% − 82.6%)
Community violence	18.6% (12.5% − 26.7%)	12.4% (8.0% − 18.7%)	40.2% (31.7% − 49.4%)

Source: the authors (2024)

CI – Confidence Interval 95%

As shown in Figs. 1 and 2, connections were found among the three types of violence at all developmental stages studied, particularly in adolescence. The categories of violence whose paths stood out for their higher coefficients and direct relations with SB/NSSI were psychological family violence in childhood (0.656, *p* = 0.006), community violence in adolescence (0.517; *p* < 0.001) and psychological family violence in youth (0.398, *p* < 0.001). Physical family violence in adolescence (-0.747, *p* = 0.001) and physical family violence in childhood (-0.402, *p* = 0.045) also proved important, but in the opposite direction, with less violence associated with more SB/NSSI.

Psychological violence in adolescence and physical violence in youth deserve attention for results that were narrowly statistically significant (*p* = 0.088). Nonetheless, there was a web of important effects at each stage of life, indicating possible relations among the types of violence: in childhood, notably between community and psychological violence (0.348, *p* < 0.001) and between psychological and physical violence; in adolescence, all the types of violence were interrelated; and, in youth, effects were found between community and psychological violence (0.302, *p* < 0.001) and between physical and psychological violence (0.374, *p* = 0.001). Psychological violence was important at the three developmental stages, as shown by significant or near-significant coefficients.

## Discussion

This study adds to longitudinal investigations into the emergence and continuance of adverse mental health outcomes among young people. It prioritised pathways among types of violence and between violence and suicidality, in the light of child and youth developmental stages, making it possible to collect data sensitive to the participants’ psycho-pathological specifications.

The discussion did not disregard the possible presence of mental disorders; however, the complexity and multi-causality of this universal phenomenon call for broader views, particularly when its occurrence is examined in vulnerable and minority groups [31, 46–48]. In these groups, an exclusively clinical approach as the sole vector in prevention has not been effective [49], recommending ecologically based strategies that address both structural and interpersonal violence. The aim, therefore, was to shed light on explanations that complement the association between mental disorders and suicidality, emphasizing universal pathways for prevention. This explains the decision not to include mental disorders as variables in the analysis.

The chosen focus here, on forms of violence suffered by young people, rests on the conviction that, because of their prevalence and connection with psychiatric conditions proper, these should be a priority for public health interventions. This draws a parallel with the proposal of Ports et al. [28] that prevention of ACEs should constitute the main measure for preventing suicide. That study proposes upstream strategies, placing emphasis on violent events, the rationale being that the path to suicidality is not always mapped by the diagnosis and/or presence of a psychiatric condition, but that it is more likely to run closer to the various different forms of violence and ACEs, which tend to find expression most critically in minority and vulnerable groups [11, 48]. The context of the participants’ vulnerability and the violence they experienced, and the current context of the American continent, with its rising numbers of deaths by suicide, justify that methodological choice [50].

From observation of the distribution of the nine violence events studied (Table 3), it was inferred that, in adolescence, violent experiences are probably less perceived. In this study, adolescence represented the first developmental stage in which the participant served as the primary informant - a factor that, when considered alongside the transitional nature of this life stage and the increased self-understanding it involves [35], may help explain the observed finding.

Three situations stood out: high frequency of family violence in childhood and community violence in youth; and the magnitude of psychological violence longitudinally. Although occurring less in adolescence, the structural equations modelling (Figs. 1 and 2) identified a path followed by psychological violence over the life course, from childhood to adolescence (*p* < 0.001) and from adolescence to youth (*p* = 0.063). That path was not a constant for the other forms of violence investigated, signalling peculiarities posed by the invisibility, naturalization and revictimization of psychological violence, as discussed by Glaser [51], and Spinazzola et al. [52].

The forms of family violence were prominent in childhood, and psychological family violence was important in youth. From that perspective, the various forms of violence constitute veritable betrayals in the microsystem, in the intimate space of the nuclear family [8, 10] – albeit in family relationships with potential affective connections, even if residual or ambivalent. The variables indicated in the literature that define the impact of ACEs are their duration, type and severity, which influence each subject’s susceptibility to violence, particularly at more sensitive and care-dependent stages of development. Particularly notable is the persistence of variables relating to violence caused by the mother, which reflect the child’s life from birth, and not just those occurring at school age. That impact also disorganises systems, modulating their resilience and protective factors [5, 15].

As ruptures on the developmental plane, ACEs demand and drive neuro-biological adaptations to assure survival and the perpetuation of the species. That adaptation, however, may entail risks expressed, over the course of life and through different ecological systems, in genetics, epigenetics and/or phenotypes. Depending on the modulation of resilience protection factors, this may be expressed through neuro-psychiatric symptoms [5, 6, 16, 17].

Despite the connection found between interpersonal and community violence and self-inflicted violence, it is important to stress that frequencies of non-suicidal self-injury, suicidal ideation and attempted suicide were lower than for the other forms of violence. This then raises the possibility of understanding severity in the spectrum of violence and that other protection and resilience factors are also acting on suicidality [46, 47].

The prominence of psychological violence in the study findings may be explained by the length of the timeframe over which it tends to be manifest: as a phenomenon that is rendered invisible, particularly in childhood, it tends to be perpetuated in future relations [51, 52]. When present in early childhood, its perpetuation can more readily shape the language and construction of disabling attachment [6, 53], predisposing to learning a pattern of violent relations, low self-esteem and emotional dysregulation. Over the life course, these symptoms predispose to suicidal or non-suicidal self-injury in adolescents and young people [9, 26, 54].

That prominence may explain, in this cohort, the reverse direction of the path of physical family violence in childhood and adolescence in relation to the outcome. It is suggested that the proximity between physical and psychological violence and, considering psychological suffering, a tendency for repercussions of psychological violence to crystallise more could be key to understanding this finding. Thus, despite acknowledging experiences of physical violence during childhood and adolescence, the analysis highlighted psychological violence in the emergence of suicidality. It is, however, acknowledged that serial studies will be important to elucidate these data better.

The findings of two recent Brazilian studies [8, 21], have also pointed to the importance of psychological violence, which may suggest new directions for investigation and intervention. This quality of invisibility and the cultural normality with which emotional abuse is sometimes regarded also account for the difficulty of actively addressing forms of community violence, such as racism and other racial and/or social discrimination.

Alvarez et al. [31], deploying the bio-ecological model to discuss how structural and community violence are established in the daily lives of minority youth, directed their attention to prevention strategies that address violence and involve family and community. From that perspective, it is easier to understand how violence leads to violence and that ACEs carry risks of co-occurrence and various risk factors for suicidal behaviour and non-suicidal self-injury. Jewett [24] also explored this thinking by discussing how structural violence disorganises and undermines social and family relations, amplifying new violence, emotional suffering, social vulnerability and suicidality.

With the model proposed here, it could be seen that, in the three stages of life, there was a relation among the types of family and community violence studied, a finding that agrees with clinical and research scenarios, where ACEs are found to overlap with the different forms of violence [22, 25, 30]. It is thus possible to demonstrate the cumulative, amplifying effect of forms of violence or ACEs, corroborating the urgency argued here of preventing all forms of violence. In this study, the connections were most evident in adolescence, suggesting that this stage continues to be critical and vulnerable; in adolescence, specifically its latter half, cases of suicidal behaviour and non-suicidal self-injury are observed to increase globally [4, 12].

Studies of the neurobiology of trauma have not yet been able to specify definitively the pathways from harm from adversities in childhood to mental disorders, but they do acknowledge suicidality to develop, directly or indirectly, from that relation [23, 25]. In addition to the increase in self-injurious behaviour, ACEs are related to earlier onset of mental disorders, poorer response to treatment and presence of more comorbidities [5, 27]. This perspective on self-injurious behavior justified the decision not to disaggregate the outcome (SB/NSSI), given the unclear boundaries between suicidal behaviour and distress, and the understanding that different forms of violence and emotional suffering may lead to self-inflicted harm, with or without suicidal ideation.

Accordingly, the greater suicidality found over the course of the lives of participants who reported events of violence can be accounted for. More than etiological or explanatory significance, it corroborates the argument that prevention of the different forms of violence can be associated with prevention of SB and NSSI [25, 28]. The findings of this study may thus inform prevention strategies for SB and NSSI and should also be investigated in other psychosocial contexts.

## Strengths and limitations

The longitudinal data, which aggregate five data collection points over nearly two decades and encompass three developmental stages, represent one of the main strengths of this study. Follow-up studies are the most sensitive strategy for observing a relational dynamic and identifying deviations from behaviour expected of the species. At this longitudinal horizon, however, one acknowledged weakness is that the childhood stage is more investigated from age six years onwards. However, as the scale gauging family violence at the first wave was discriminated from birth, part of the violence suffered by children in the home could be introduced.

In this study, the analysis included weighting, but not the sampling plan, which is a methodological limitation. It was attempted to use a sampling plan, but problems of convergence prevented its inclusion as a strategy for correcting the interval estimates. Observed limitations in model adjustment suggest including more variables on family and community violence, highlighting the need for further exploration. Also, the model did not comprehensively address sexual violence, which should be the prioritized in future investigations to broaden the understanding of violence beyond the forms examined in this study.

Furthermore, in relation to the quality of the model obtained, the SRMR met the cutoff, suggesting that the mean standardized residuals are within acceptable limits. However, the RMSEA indicates a higher-than-ideal error of approximation per degree of freedom and the CFI remained below, revealing that the model moderately explains the observed covariance. These findings may reflect the relatively small sample size, the use of a complex study design, and the complexity of the construct. Future studies, possibly based on a different theoretical model, could include additional factors such as attachment-related vulnerabilities, parental supervision, and individual characteristics like temperament, genetics, and epigenetics.

Another point is that the study did not structure the measures by type of ACE, which difficult the comparison with important studies in this field. Given that ACEs are expressions of violence in childhood and adolescence, this study understands violence and ACEs as overlapping concepts The greater focus on violence, rather than on ACEs, was a methodological choice justified by the number of available variables relative to the sample size, as well as by the need to continue investigating the independent variables in the population over 18 years of age. Also, the frequency of mental disorders was not considered since a priority was made of seeking a comprehensive pathway between forms of violence and suicidality [23, 27]. The option for an aggregate outcome (suicidal ideation, attempted suicide, and non-suicidal self-injury) is potentially limitation when interpreting the data and should be taken into consideration, particularly when these are transposed to clinical practice. Future research should address these constructs separately, allowing for more nuanced understandings of self-injury and violence.

## Conclusions

The findings of this study add to recent evidence of the impact of violence against children and adolescents over their life course. The various adversities experienced in childhood, which are generally co-occurring and tend to have prospectively cumulative effects, alter child and adolescent trajectories, albeit by mechanisms not fully understood.

Psychological violence was notable for its invisibility, penetration and cultural tolerability, thus demanding attention from the various professions that provide care for children and adolescents. A violent family environment is a risk for suicidality, highlighting the need for care and protection to ensure safety and trust for children and adolescents.

Studies on suicidal behavior and self-injury must progress toward an open, democratic dialogue with society to challenge taboos and foster a relational system that protects youth [31, 49]. Simultaneous “cohorts” of young people around the world, particularly in developing countries, are in distress, and it is urgent to propose multiple approach strategies, research techniques and mental health promotion measures. The data presented here can collaborate towards new strategies for recognising and combating violence as a critical mental health promotion and prevention strategy.

## Data Availability

No datasets were generated or analysed during the current study.

## Abbreviations

WHO: World Health Organizations
ACE: Adverse Childhood Experiences
PAR: Population Attributable Risk
SB: Suicidal Behaviour
NSSI: Non-suicidal Self-injury
ASR: Adult Self Report
ASEBA: Achenbach System of Empirically Based Assessment
YRBSS: Youth Risk Behavior Survey Scale
IMV: Integrated Motivational-Volitional
CTS: Conflict Tactics Scale
PsyFV_C: Psychological family violence in childhood
PsyFV_A: Psychological family violence in adolescence
PsyFV_Y: Psychological family violence in youth
PhyFV_C: Physical family violence in childhood
PhyFV_A: Physical family violence in adolescence
PhyFV_Y: Physical family violence in youth
ComV_C: Community violence in childhood
ComV_A: Community violence in adolescence
ComV_Y: Community violence in youth
